# Macro–Micro Properties of Remodeled Waste Slurry Under Freeze–Thaw Cycles

**DOI:** 10.3390/ma18010178

**Published:** 2025-01-03

**Authors:** Long Wang, Houren Xiong, Junguang Huang, Minjie Wen, Pan Ding, Yiming Zhang

**Affiliations:** 1 School of Civil and Transportation Engineering, Hebei University of Technology, Xiping Road 5340, Tianjin 300401, China; wanglong199801@163.com (L.W.); 202111601004@stu.hebut.edu.cn (J.H.); 2 School of Civil Engineering and Architecture, Jiaxing University, Jiaxing 314000, China; xionghr2018@zjxu.edu.cn; 3 School of Civil Engineering and Architecture, Zhejiang Sci-Tech University, Hangzhou 300018, China; 0620577@zju.edu.cn (M.W.); dingpan@zstu.edu.cn (P.D.)

**Keywords:** engineering waste slurry, road pavement, freeze–thaw cycles, microstructure, macro–micro response

## Abstract

Waste slurry, a major by-product of urban construction, is produced in rapidly increasing volumes each year. Dehydrated waste slurry has potential as a roadbed material; however, its performance in freeze–thaw environments, which can induce frost heave and thaw settlement, and the mechanism of the influence of freeze–thaw cycles on its macro and micro properties are still unclear and need thorough investigation. This study explores the macroscopic and microscopic properties of waste slurry subjected to freeze–thaw cycles. We conducted unconfined compressive strength (UCS) and triaxial unconsolidated undrained (UU) shear tests, focusing on fissure compaction, elastic deformation, plastic yielding, and strain hardening stages. The results reveal a decrease in strength and elastic modulus with increasing freeze–thaw cycles, as well as in the damage degree generated by freeze–thaw cycles. To uncover the underlying microscopic mechanisms, we performed Scanning Electron Microscopy (SEM), X-ray diffraction (XRD), and mercury intrusion porosimetry (MIP) analyses. These tests highlighted the evolution of pores and microcracks during freeze–thaw cycles. These results have important reference values for the reutilization of waste slurry discharged from large-diameter bored piles for roadbed backfill materials that need to be repaired quickly in seasonally frozen areas.

## 1. Introduction

The emission of engineering waste slurry has reached a global record high. This phenomenon is especially prominent with the rapid development of China’s railways [1], the annual volume of which has exceeded 225 million m^3^ [2,3]. Inappropriate disposal of waste slurry can cause environmental pollution and land waste [4,5], and burning waste slurry into bricks and ceramics consumes energy and produces harmful gasses [6,7,8]. Researchers have proposed other effective and safe procedures for recycling waste slurry [9,10]. For example, after the pre-treatment of waste slurry, various additives are added to produce construction materials, replacing ordinary sand and stone materials in the construction of road subgrade [11]. When using treated waste slurry to construct road subgrade, environmental impacts such as temperature fluctuations, which may cause frost heave and thaw settlement of roads, should be considered [12].

Freeze–thaw cycling causes cyclic phase changes in water in porous media and the subsequent deterioration of the mechanical properties of waste slurry. Considering soil materials, Zhou et al. [13] and Tang et al. [14] found that the Young’s modulus and tensile and shear strengths of the soil were significantly reduced under several freeze–thaw cycles. Hotineanu et al. [15] found that the cohesion of expanded clay soil gradually decreased with the increase in freeze–thaw cycles, while the friction angle slightly increased. Yang et al. [16] investigated the effect of freeze–thaw on the dynamic properties of soil under different boundary pressures. They found that high boundary pressures induced soil consolidation during the freeze–thaw process. The deterioration in the engineering properties of the soil under freeze–thaw cycles can be revealed by investigating its microstructure [17,18]. Using mercury intrusion porosimetry (MIP) and X-ray computed tomography tests, Tang et al. [19] found that the porosity of the soil increased, and the fractal dimension slightly decreased under freeze–thaw cycles. Li et al. [20] investigated the deformation characteristics and microstructural features of the soil under different loads and found a loading law that makes the volume of the soil constant during freeze–thaw cycles.

In practical engineering applications, Su et al. [3]. modified and upgraded the waste slurry residue into subgrade filler and evaluated the stability of the reinforced site on the temporary road around the south extension project of Heping Avenue in Wuhan, China. The construction material of the road subgrade and subbase was SWGA-BF improved waste slurry, and the measured results of CBR were both greater than 6%. This met the requirements of JTG D50-2017 and JTG D30-2015. However, the influence of freeze–thaw environment in different regions on the backfilling of waste slurry materials in subgrade was not considered.

The above research results on engineering waste slurry serve as the background for this paper; however, the mechanism of the influence of freeze–thaw cycles on the macro and micro properties of slurry is still unclear and needs further study. Therefore, the macro- and microstructure analysis methods were used for in-depth analysis. The global optimization method was innovatively introduced, and the relationship between the macro and micro properties of waste slurry was established by combining SEM, MIP, and ImageJ-v1.53a software. This study took waste slurry generated from large-diameter bored piles at an intercity railway construction site for investigation. The waste slurry was firstly dehydrated and then wetted to its optimal moisture content. In macroscopic studies, the effects of different numbers of freeze–thaw cycles on the mechanical properties of the waste slurry were systematically analyzed by a UU test and UCS test. In microscopic studies, SEM, XRD, and MIP tests were conducted, showing the changes in pores and microcracks in the waste slurry with freeze–thaw cycles. Pearson’s correlation method was used to obtain the correlation between macro- and micro-parameters, showing strong correlations among strength, skewness, shape abundance, MIP permeability, and porosity. The quantitative relationship between macro- and micro-parameters was established.

## 2. Materials and Methods

### 2.1. Waste Slurry

**Figure 1:**
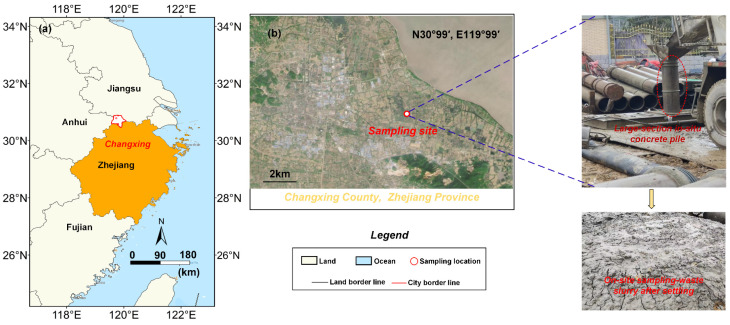
(**a**) The geographical location of the project from which the waste slurry was sourced; (**b**) satellite photographs.

**Figure 2:**
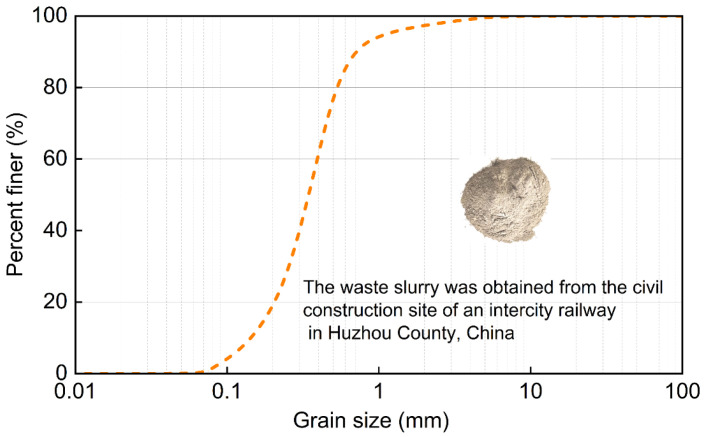
Grain size distribution of waste slurry.

Waste slurry was obtained from the civil construction site of an intercity railway in Huzhou County, China (N 30°99′, E 119°99′), The average annual temperature is 15.6 °C, the temperature varies between ±0.5 and 0.7 °C, and the inter-annual temperature range is 1.2 °C. as shown in Figure 1. This slurry is the waste discharged from the construction of large-diameter bored piles in composite strata with muddy silty clay and silty sand. The test material was light gray waste slurry. Indoor tests were conducted according to ASTM D4318: Atterberg, Casagrande [21,22], and the test results are summarized in Table 1. The grain size distribution test results are shown in Figure 2.

**Figure 3:**
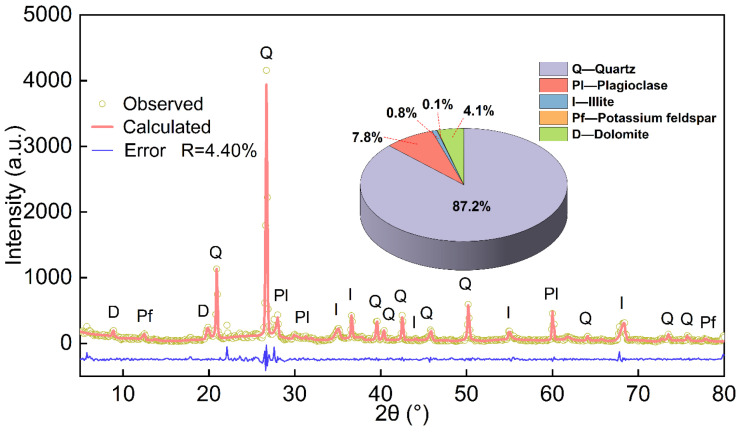
X-ray diffraction patterns.

The main components of the slurry are quartz (87.2%), plagioclase (7.8%), dolomite (4.1%), illite (0.8%), and potassium feldspar (0.1%), obtained by XRD as shown in Figure 3. SEM identified three primary types of microstructures in the engineering waste slurry from this site: honeycomb structure, coagulated block structure, and flocculent structure. More details of the microscopic structures of the slurry can be found in Section 3.3, Section 3.4 and Section 3.5.

### 2.2. Specimen Preparation

**Figure 4:**
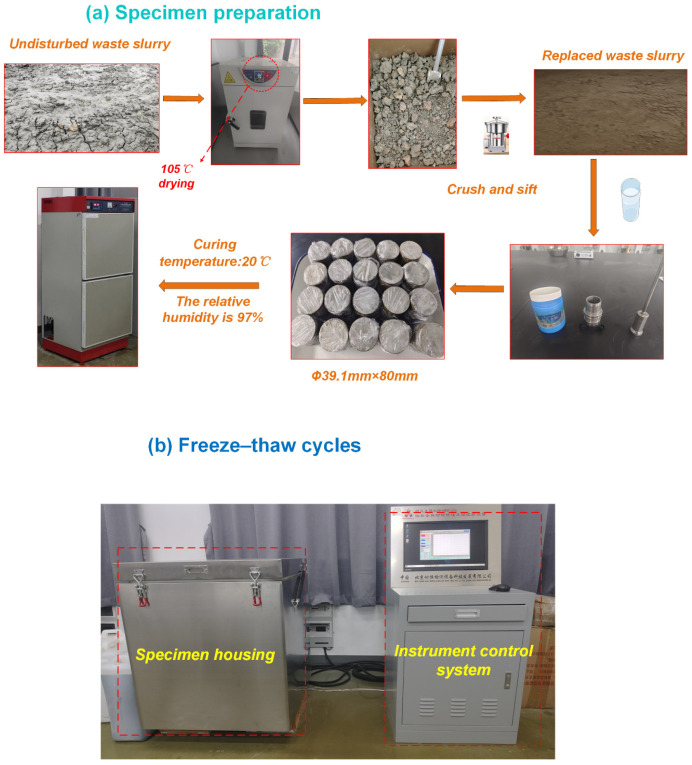
Procedure of specimen preparation and freeze–thaw test equipment.

The diameter of the sample was 39.1 mm and the height was 80 mm [17,18]. The steps for sample preparation were as follows: Firstly, the waste slurry was dried at 105 °C and crushed, and it was then filtered through a 2 mm sieve, as shown in Figure 4. Secondly, a predetermined amount of distilled water was added to the material, which was allowed to stand for 15 min to achieve complete absorption, ensuring the same optimum moisture content for the remolded waste slurry, as shown in Table 1. Finally, the remolded slurry was poured into a mold and compacted with vibration. After the specimens were prepared, they were kept in a standard curing box with a temperature of 20 °C ± 1 °C and relative humidity greater than 95% for 28 days [23].

### 2.3. Freeze–Thaw Cycle Test

**Figure 5:**
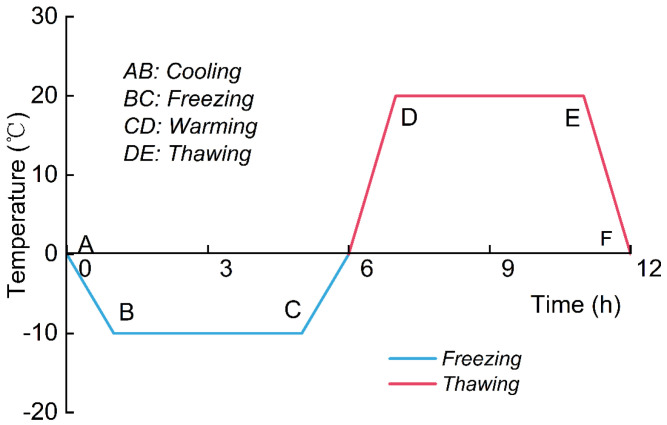
Temperature–time curve during FT cycles.

The damage degree of the specimen caused by the freeze–thaw cycle is mainly affected by three factors: the duration of each cycle, the freeze–thaw temperatures, and the number of cycles [24,25]. The duration and the freeze–thaw temperatures are commonly determined by historical temperatures. The temperature–time curve was considered according to the climate records of Changxing City, Zhejiang Province, as shown in Figure 5; the freezing temperature was −10 °C and the thawing temperature was 20 °C. Each cycle was set to 12 h with 4 h freezing and 4 h thawing. This work considers 5 cycle numbers: 0, 3, 6, 9 and 12.

### 2.4. UU Test

**Figure 6:**
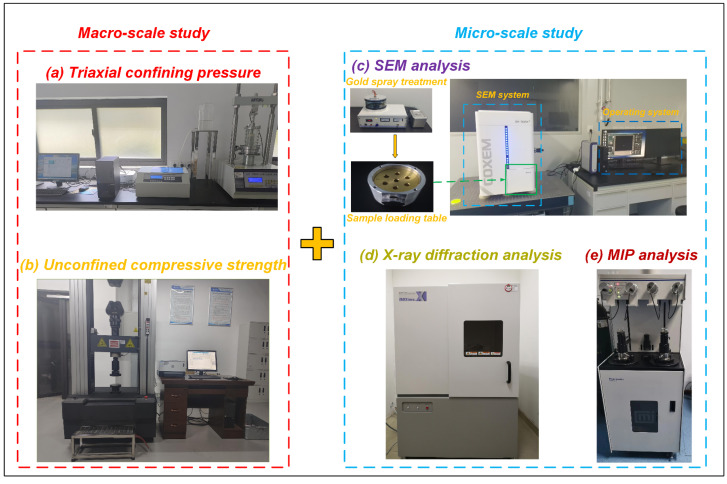
Macro and micro test equipment flow.

The fully automated STSZ-ZD triaxial testing machine shown in Figure 6a was used to conduct the UU triaxial test of the remodeled slurry waste. Six specimens were prepared for each number of freeze–thaw cycles. Three peripheral pressures of 0.1, 0.2, and 0.4 MPa were used. Pressure was increased from 0 to a predetermined value at a rate of 0.5 MPa/min. After applying the circumferential pressure, the displacement-controlled axial pressure was applied with a rate of 1 mm/min. The test was stopped when the axial strain reached 20%.

### 2.5. UCS Test

The UTM5305 microcomputer-controlled electronic universal testing machine, shown in Figure 6b, was used for the UCS tests. In the UCS test, the specimens were kept warm after the freeze–thaw cycle. The cling film was torn off and the filter paper was removed. The displacement-controlled loading rate was 1.5 mm/min.

### 2.6. SEM Test

The microstructural analysis of waste slurry using SEM involves several essential steps: specimen preparation, pre-treatment, imaging, digital image processing, and micro-parameter calculation. In this study, as shown in Figure 6c, SEM images of the waste slurry specimens were captured using an EM-30AX+ type SEM. The specimens were prepared with dimensions of approximately 5 × 5 × 5 mm. To prevent any structural changes, the specimens were thoroughly dried immediately before SEM analysis. They were then mounted on a stage using conductive paste, followed by a gold sputtering process to enhance conductivity. Once prepared, the specimens were placed in the SEM chamber, where appropriate magnification levels and representative fields of view were carefully selected for imaging [26].

### 2.7. XRD Test

The XRD test was carried out using a Shimadzu XRD-7000 X-ray diffractometer, as shown in Figure 6d. Before the test, small pieces of specimens that had not been subjected to freeze–thaw cycles and those that had been subjected to freeze–thaw cycles were ground to a powder and then, after drying at 45 °C, placed in the diffractometer for the test to investigate the changes in the composition of the minerals before and after the freeze–thaw process. The scanning angle range was 2.4°~45°, and the scanning speed was 6°(2θ)/min. The “K value” method in SY/T 5163-2018 was used to quantitatively analyze the mineral composition [27]. The error of quantitative analysis of the minerals in Figure 3 is 4.40%

### 2.8. MIP Test

The MIP test was carried out using the AutoPoreV mercury intrusion porosimetry analyzer, as shown in Figure 6e. The working principle is to use the non-wettability of mercury to solids to make mercury enter the pore space under the action of an external force, and the greater the pressure, the smaller the pore diameter that mercury can enter. Therefore, pore volume can be characterized by measuring the amount of mercury that enters the pore space at different external pressures. According to Washbum’s equation [28], the relationship between the pressure value and the amount of liquid mercury consumed can be obtained, thus determining the pore volume of the specimen according to Equation (1).

(1) $$r=\frac{-2\gamma \cos\alpha }{P}$$

In the above equation,  r is the pore radius (μm), γ is the surface tension of mercury (N/m), α is the contact angle between the solid and mercury (°), and P is the external force applied (Pa).

## 3. Results and Discussion

### 3.1. Stress–Strain Responses of UU Test

**Figure 7:**
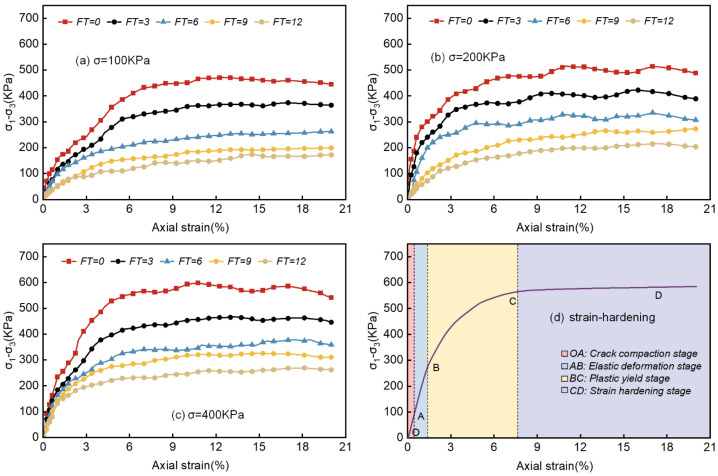
Stress–strain curves and typical strain-hardening stages of different freeze–thaw times under the same confining pressure.

The stress–strain curves for varying freeze–thaw cycles under consistent pressure conditions were plotted with the principal stress difference ($\sigma_{1}-\sigma_{3}$) on the vertical axial and axial strain on the horizontal axial, as illustrated in Figure 7. The curves exhibit strong similarity across different freeze–thaw cycles, all reaching peak stress at approximately 5% to 10% axial strain. Additionally, the peak strength increases as circumferential pressure rises. While stress increases with strain, the rate of increase gradually slows, eventually approaching an asymptote, indicating distinct strain-hardening behavior.

The degree of strain hardening in the waste slurry increased with peripheral pressure, consistent with the findings of Yang et al. [16]. Additionally, as freeze–thaw cycles increased, the stress–strain curve shifted downward and to the right, indicating a reduction in peak strength. The stress–strain curve can be divided into four distinct stages (Figure 7d): the fracture compaction stage (OA), the elastic deformation stage (AB), the plastic yielding stage (BC), and the strain hardening stage (CD) [29]. In the OA stage, during the initial application of triaxial circumferential pressure, the axial load causes the original holes and small cracks within the waste slurry specimen to close. In the AB stage, the stress–strain relationship approximates a linear trend; the specimen exhibits no significant cracks, and the initial small internal cracks are compacted, leading to more rapid stress growth and minimal deformation. During the BC stage, new small cracks appear, and existing cracks develop further, resulting in irreversible plastic deformation. Here, strain increases faster than stress, causing the stress–strain to become upwardly convex as stress reaches its peak. In the CD stage, the internal particles of the specimen bond closely together. The material’s plasticity, combined with the surrounding pressure, allows the specimen to continue loading. Consequently, strain increases while stress remains relatively constant.

The Moore–Coulomb strength criterion is a classical strength criterion in rock mechanics which can accurately predict the strength of waste slurry. According to this theory, the failure of waste slurry is mainly caused by shear failure and depends on the maximum principal stress $\sigma_{1}$ and the minimum principal stress $\sigma_{3}$. The reason for adopting the Moore–Coulomb strength criterion for analysis in this paper is that according to the failure mode above, most of the waste slurry experiences shear failure under confining pressure, which agrees with the Moore–Coulomb shear failure theory.

**Figure 8:**
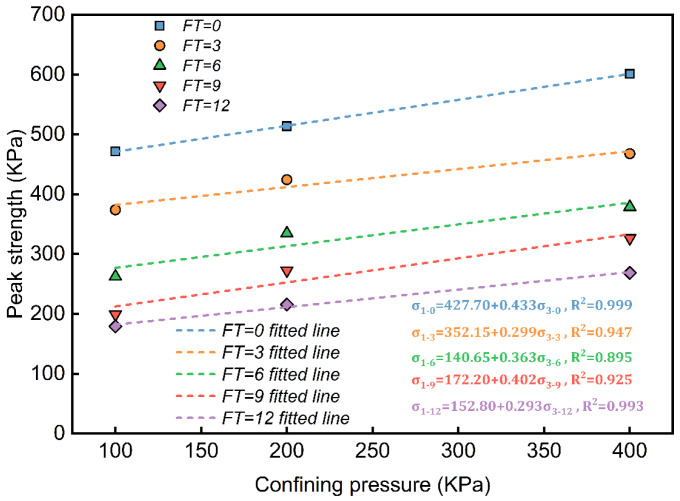
The relationship between the peak strength of engineering waste slurry and confining pressure under different freeze–thaw cycles.

Figure 8 shows the impact of enclosure pressure and the number of freeze–thaw cycles on the peak strength of the waste slurry. Peak strength decreases with an increasing number of freeze–thaw cycles, although the rate of decrease slows progressively. Regression analysis of the relationship between the peak strength and enclosure pressure yielded highly satisfactory linear results, with correlation coefficients of ${R_{0}}^{2}=0.999$, ${R_{3}}^{2}=0.947,{R_{6}}^{2}=0.895,{R_{9}}^{2}=0.925,{R_{12}}^{2}=0.993$. The fitting effect was good, and the function requirement between shear strength and normal stress in the Mohr–Coulomb criterion was met.

**Figure 9:**
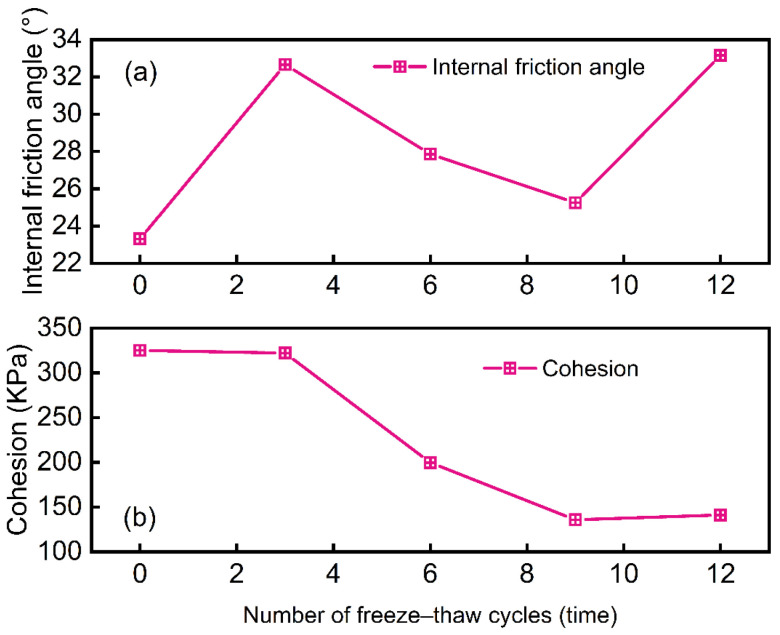
(**a**) The relationship between the cohesion and (**b**) internal friction angle of the waste slurry and the number of freeze–thaw cycles.

Figure 9 illustrates the relationship between the cohesion and internal friction angle of the waste slurry and the number of freeze–thaw cycles. The internal friction angle complex changes; specifically, as shown in Figure 9a, for freeze–thaw cycles 3, 6, 9, and 12, the changes compared to the initial state are 40.11%, 15.52%, 8.32%, and 42.21%, respectively. This indicates a wave-like pattern, with increases followed by decreases and then increases again. Figure 9b demonstrates that the cohesion of waste slurry generally decreases with the number of freeze–thaw cycles [15]. The reduction in cohesion is 0.92%, 38.55%, 58.21%, and 56. 57% after 3, 6, 9, and 12 cycles, respectively. The rate of decrease is smallest between cycles 0 and 3 and the largest between cycles 3 and 6.

The cohesion decreases least between cycles 0 and 3, decreasing most between cycles 3 and 6, and then slows after cycle 6, with a slight increase between cycles 9 and 12. This behavior can be attributed to the expansion of water as it freezes during the early stage of the freeze–thaw cycle. In the early stages, the specimen’s structure is disrupted by the internal pressure from ice formation, leading to the destruction of mineral particles and an increase in particle spacing, which reduces cohesion. By the sixth freeze–thaw cycle, visible macroscopic cracks form, further decreasing cohesion.

### 3.2. Unconfined Compressive Strength and Modulus of Elasticity

**Figure 10:**
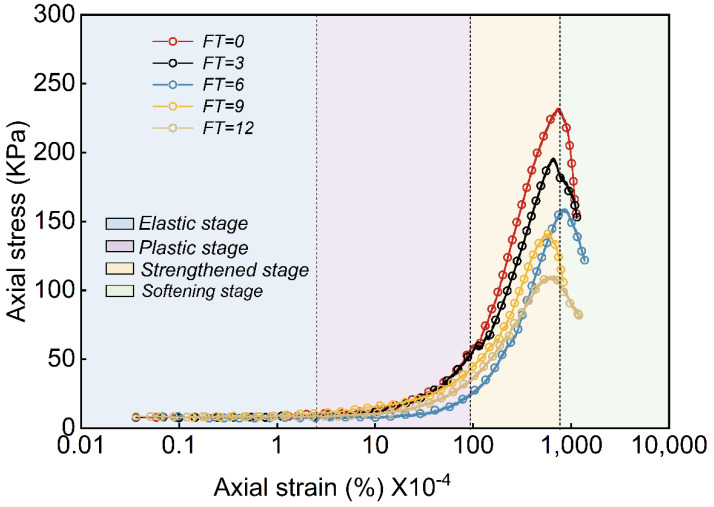
Stress–strain curve of the whole process of engineering waste slurry under freeze–thaw action.

Figure 10 displays the unconfined full-process stress–strain curves of the waste slurry subjected to varying numbers of freeze–thaw cycles. The results show that the stress–strain relationship exhibits a characteristic fracture behavior. Initially, during loading, cracks within the waste slurry close due to compression, with a gradual transition from elastic to plastic deformation. As loading continues, deformation becomes more pronounced relative to stress growth, and a typical “yield platform” emerges, indicating the slurry is in the plastic yielding stage, the stress–strain curve progresses from yielding to strengthening, marked by localized fractures that expand and connect to form a sliding shear surface. At this point, the strength reaches its peak, known as the unconfined compressive strength ($Q_{u}$) of waste slurry. Beyond the peak, the slurry rapidly fails, with strength rapidly reduced while axial strain remains relatively small and lateral strain continues to increase. The stress–strain curve then exhibits a “hump-shaped” pattern, reflecting pronounced strain-softening characteristics [30].

**Figure 11:**
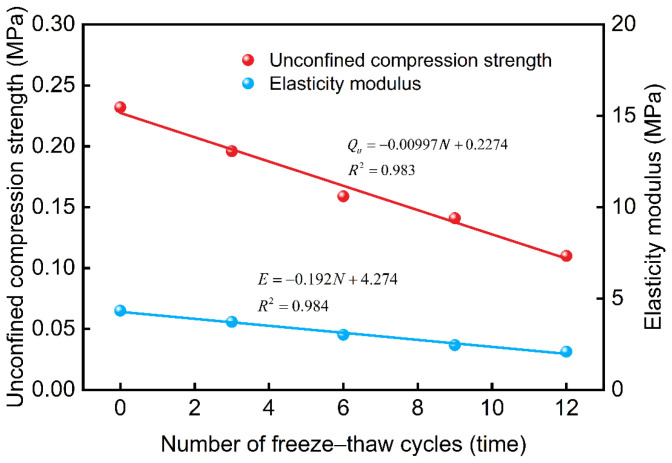
The relationship of unconfined compressive strength and elastic modulus with the number of freeze–thaw cycles.

The freeze–thaw cycle has a significant impact on the mechanical properties of waste slurry, particularly the unconfined compressive strength ($Q_{u}$) and modulus of elasticity (E), which are essential indicators of its mechanical behavior. Figure 11 shows the relationship between the unconfined compressive strength and modulus of elasticity of the waste slurry specimen with respect to the number of freeze–thaw cycles. The results show that with an increasing number of freeze–thaw cycles, both the unconfined compressive strength and modulus of elasticity of waste slurry exhibit a linear decay. Specifically, the unconfined compressive strength decreases by 15.52%, 18.88%, 11.32%, and 21.98% after 3, 6, 9, and 12 freeze–thaw cycles, respectively. Concurrently, the modulus of elasticity shows reductions of 11.07%, 19.04%, 18.66%, and 14.54% for the same cycle numbers. These findings suggest that a higher number of freeze–thaw cycles leads to increased internal damage within the waste slurry, thereby deteriorating its macroscopic mechanical properties.

### 3.3. Freeze–Thaw Damage Evolution

**Figure 12:**
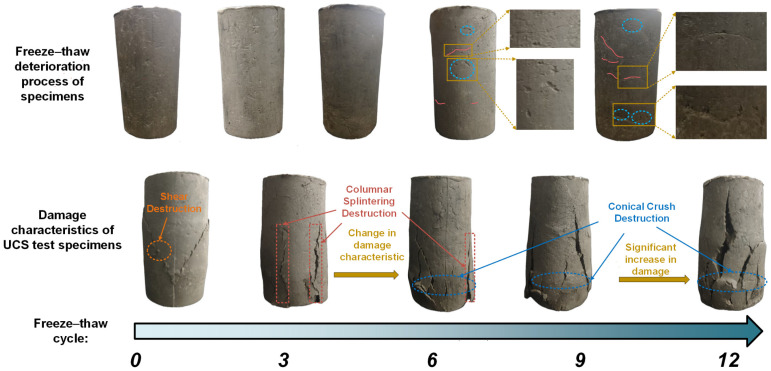
Apparent characteristics of specimens after FT and damage characteristics after UCS test.

Figure 12 shows the visible damage characteristics of the specimen after freeze–thaw cycles and subsequent UCS tests. Repeated freeze–thaw cycles led to significant internal damage, manifesting as numerous macroscopic cracks of varying sizes and orientations on the specimen’s surface (indicated by red lines), as well as the exfoliation of fine particles (indicated by blue ellipses). As the number of freeze–thaw cycles increases, the damage mode transitions from shear failure to columnar splitting and conical crushing.

The deterioration observed on the specimen surface, as depicted in Figure 12, shows that freeze–thaw damage initially accumulates rapidly. In the early stages, internal pores and fissures develop due to the expansion of freezing water, which creates pressure within the confined specimen. This pressure exceeds the constraints of surrounding particles, leading to the formation of pores and fissures. As these defects grow, they interconnect and form macroscopic cracks once the number of cycles reaches six. During this phase, water migrating through these cracks concentrates the freeze–thaw expansion forces, exacerbating the damage. In later stages, although the crack development rate decreases rapidly, the existing cracks accommodate the volume expansion, reducing the overall impact of freeze–thaw cycles. Consequently, the surface characteristics of specimens between 9 and 12 freeze–thaw cycles exhibit less variation.

To assess the freeze–thaw resistance of waste slurry more effectively, this study introduces the freeze–thaw coefficient ($K_{f}$). This freeze–thaw coefficient is defined as the ratio of the unconfined compressive strength of the slurry specimen subjected to freeze–thaw damage to the unconfined compressive strength of the specimen before freeze–thaw cycles, within a temperature range of −10 °C to 20 °C [31]:

(2) $$K_{f}=\frac{{\bar{Q}}_{f}}{{\bar{Q}}_{s}}$$

The above equation illustrates the freeze–thaw coefficient of the waste slurry; ${\bar{Q}}_{f}$ is the mean value of the unconfined compressive strength after the freeze–thaw cycle test; ${\bar{Q}}_{s}$ is the mean value of the unconfined compressive strength of the waste slurry specimens before freeze–thaw.

**Figure 13:**
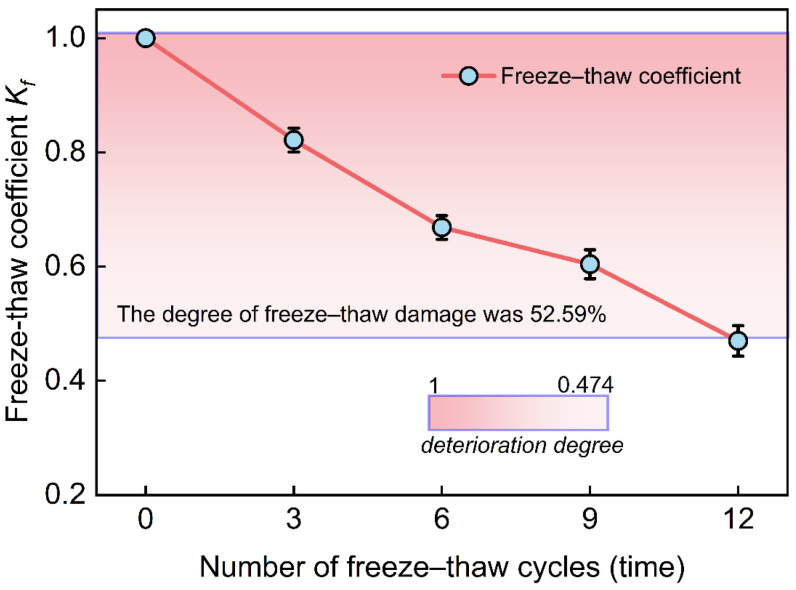
The relationship between the freeze–thaw coefficient of engineering waste slurry and the number of freeze–thaw cycles.

Based on the test results presented the calculation of Equation (2), the relationship between the freeze–thaw coefficient and the number of freeze–thaw cycles can be approximate straight line, a higher freeze–thaw coefficient indicates a greater sensitivity of the freeze–thaw cycles. As illustrated in Figure 13, after 12 freeze–thaw cycles, the coefficient decreases by 52.59%, demonstrating that the mechanical properties of the waste slurry deteriorate significantly with increased freeze–thaw cycles.

### 3.4. SEM Image Analysis and Quantitative Analysis

#### 3.4.1. Microstructural Observations

**Figure 14:**
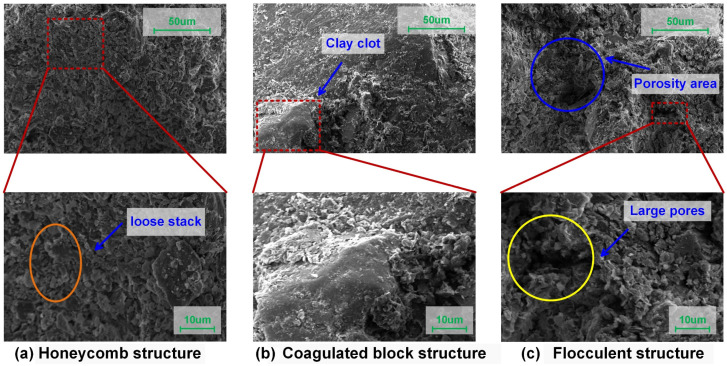
Common microstructure types of engineering waste slurry.

**Figure 15:**
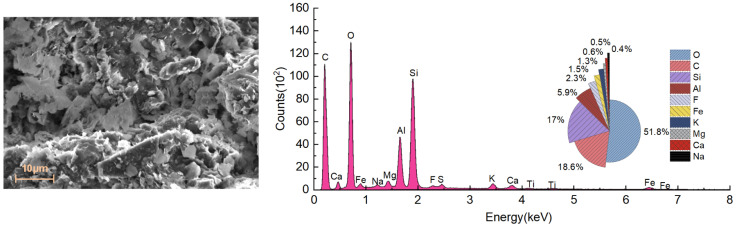
Energy dispersive X-ray spectrometry of waste slurry.

The microscopic characteristics of waste slurry were analyzed using SEM and an energy-dispersive spectrometer, as shown in Figure 14 and Figure 15. The analysis of images from SEM (magnification of 1000, 3000) identified three primary types of microstructures in the engineering waste slurry from this site: honeycomb structure, coagulated block structure, and flocculent structure.

#### 3.4.2. Evolution of Microstructure Damage in Waste Slurry 

**Figure 16:**
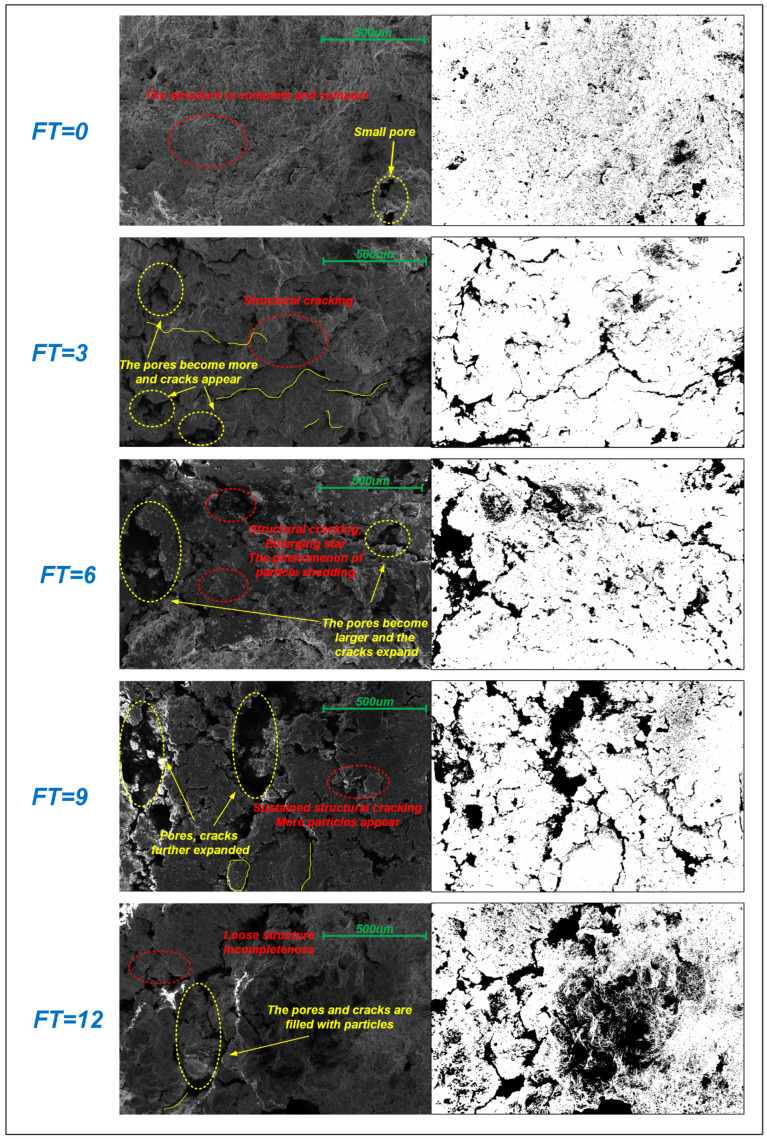
SEM images and binary denoising SEM images of engineering waste slurry under freeze–thaw cycles.

To further investigate the microstructural damage characteristics of waste slurry under freeze–thaw cycles, this study utilizes SEM combined with ImageJ software [26]. SEM images were processed using ImageJ’s advanced graphic functions. Initially, these images were converted from grayscale to a binary format, where white represents particles and black represents pores [32], as shown in Figure 16. This binarization enhances the visualization of cracks, pores, and particles, providing a clearer understanding of the material’s microstructure. The analysis revealed that freeze–thaw cycles significantly affect the waste slurry’s microstructure, including changes in pore area, diameter, particle abundance, and fractal dimension. These factors collectively elucidate the complex micromechanical behavior of the waste slurry [33].

Figure 16 shows microscopic images of the waste slurry under multiple rounds of freeze–thaw cycles with a magnification of 500. In the absence of freeze–thaw cycles, the mineral particles in the waste slurry were well-preserved with a relatively intact structure. However, after three freeze–thaw cycles, the cracks between the particles widened, and the structure began to fragment locally, increasing porosity. With additional freeze–thaw cycles, the surface particles became dislodged, and the pores and cracks expanded and merged, resulting in more extensive disintegration. This deterioration is primarily attributed to the thermophysical properties of the waste slurry and the water-ice interactions. As the temperature drops, structural cracks form due to temperature-induced damage. During freezing, the expansion of ice further exacerbates these cracks. Conversely, during thawing, the reduction in expansion forces allows meltwater to penetrate internal pores or capillary channels, setting the stage for further damage in subsequent freeze–thaw cycles. Over time, this repeated process leads to significant crack formation and structural fracture. The waste slurry, initially compromised, becomes increasingly vulnerable to severe weathering damage under continuous freeze–thaw cycling, which reduces its stability.

After six freeze–thaw cycles, surface cracks deepened, and small particles began to detach. By nine freeze–thaw cycles, cracks between the waste slurry particles had widened, local fragmentation intensified, and pore development became more pronounced. Continued freeze–thaw cycling led to progressively looser surfaces. with additional particle detachment and pore and crack filling.

#### 3.4.3. Effects of Freeze–Thaw Cycles on Pore Characteristics

**Figure 17:**
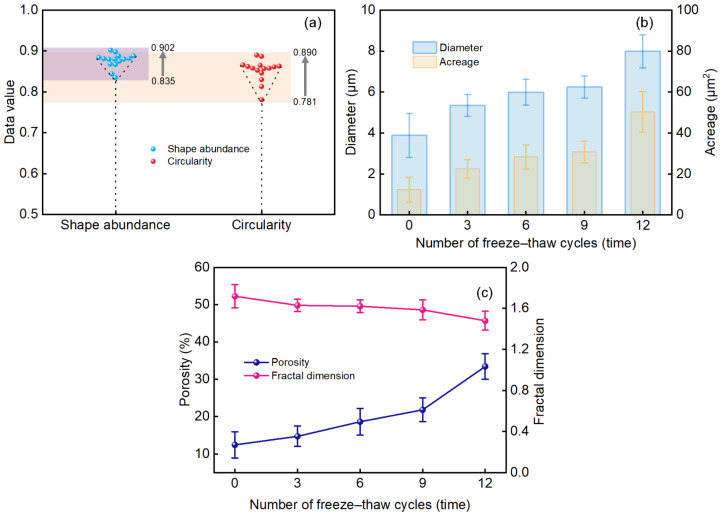
(**a**) Pore shape abundance and roundness distribution. (**b**) The changing law of pore diameter and area. (**c**) The changing law of porosity and fractal dimension.

The microscopic parameters of the waste slurry pores were analyzed using ImageJ, as illustrated in Figure 17. The results in Figure 17a reveal that the shape of the waste slurry pores varies from 0.835 to 0.902, indicating that the difference between the short and long axes of the pores is relatively minor. Additionally, the pore circularity ranges from 0.781 to 0.890, suggesting that most pore spaces are nearly circular. Figure 17b illustrates that both the diameter and area of the waste slurry pores increase with the number of freeze–thaw cycles. The growth rate is initially higher between cycles 0 and 3 but then declines. However, after nine cycles, the growth rate accelerates, indicating ongoing pore expansion.

Figure 17c shows that the distribution of particle morphology, fractal dimension, and porosity changes with the number of freeze–thaw cycles. The fractal dimension, which measure the complexity of particle morphology, decreases as the number of freeze–thaw cycles increases. This decreasing trend suggests that with more cycles, the pore area enlarges and debris particles become more separated, reducing the particles’ complexity or roughness. The porosity data indicate an accelerated expansion of pores and cracks, visible at the microscopic level, and an increase in internal damage with additional freeze–thaw cycles.

### 3.5. Analysis of XRD Results

**Figure 18:**
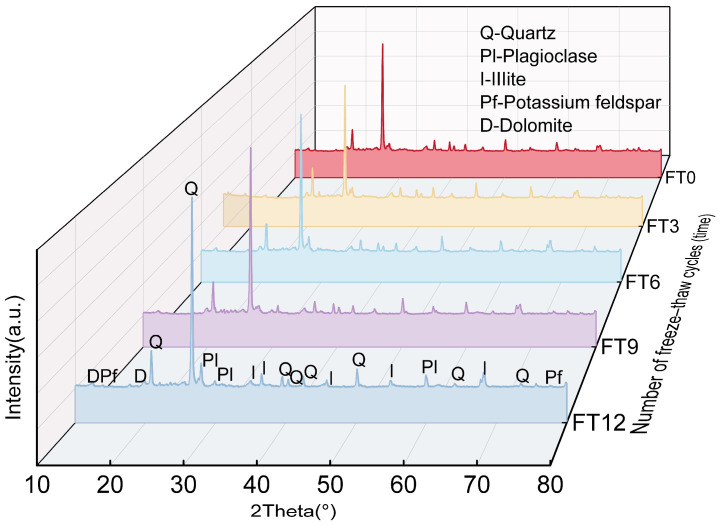
The XRD diffractograms of the waste slurry after various freeze–thaw cycles.

Figure 18 shows the XRD diffractograms of the waste slurry after various freeze–thaw cycles. The data indicate a slight increase in the proportion of quartz, while the proportions of plagioclase feldspar, illite, potassium feldspar, and dolomite slightly decreased. However, no new compounds were formed, and the overall mineral composition remained unchanged.

Quartz, due to being chemically stable, is not prone to hydrolysis during freeze–thaw cycles. In contrast, other minerals, which are hydrophilic, undergo dissolution and hydrolysis during these cycles. Clay minerals such as plagioclase feldspar and ilmenite swell unevenly after absorbing water, leading to the formation of microcracks. The infiltration of water into micro-fissures facilitates the combination of hydrophilic minerals with water, resulting in the formation of hydrated clay. This hydrated clay exhibits characteristics such as expansion, rheology, dispersion, and flocculation. Consequently, freeze–thaw cycles accelerate the disintegration of clay minerals, thereby increasing the porosity of the waste slurry.

### 3.6. Waste Slurry Pore Throat Evolution

**Figure 19:**
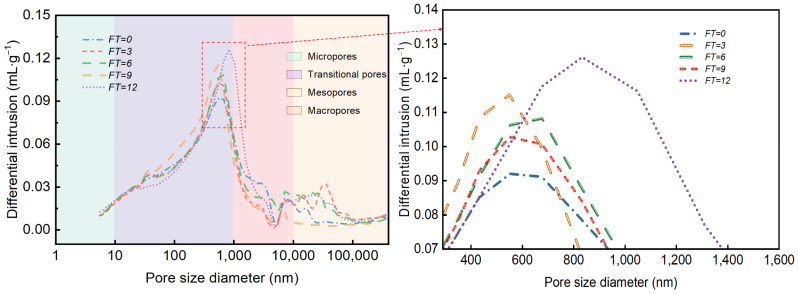
Pore size distribution diagram of engineering waste slurry.

The pore structure of waste slurry consists of pores and pore throats. Pores serve as storage spaces for fluids in porous materials, while pore throats act as connecting the pores, facilitating fluid flow. The presence and characteristics of pore throats significantly influence the porosity and permeability of the waste slurry [34]. To quantitatively characterize the evolution pore structure in waste slurry specimens subjected to freeze–thaw cycles, pore throat size distribution was analyzed using size distribution curves. The pore throats in waste slurry (Figure 19) were classified into four types: micropores (e<10nm), transition pores (10nm≤e<1000nm), mesopores (1000nm≤e<10,000nm), and macropores (10,000nm≤e<100,000nm). The pore throat size distribution curves reveal that pore throats in the waste slurry range from 10nm≤e<10,000nm, with the majority (10nm≤e<1000nm) being transition pores, representing 65% to 87% of the pore structure across different freeze–thaw cycles. Micropores (e<10nm) constitute the smallest proportion.

**Figure 20:**
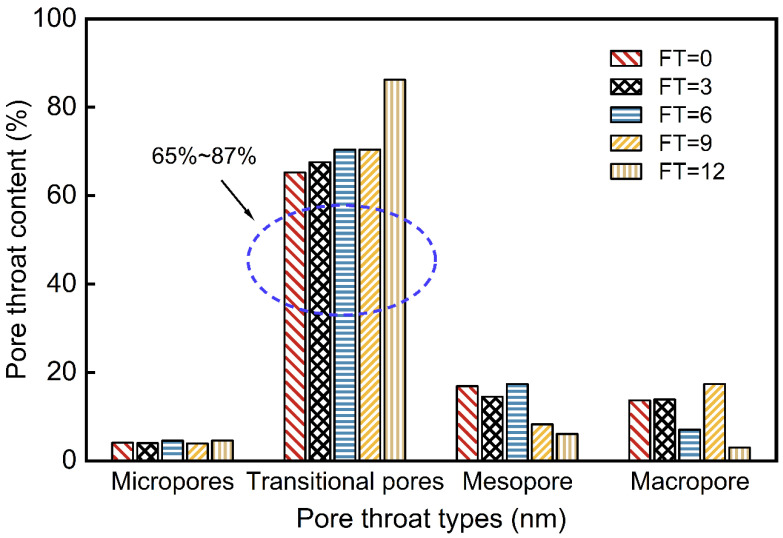
Evolution of pore throat size distribution of engineering waste slurry under different freeze–thaw cycles.

To characterize the various sizes of pore throats in the waste slurry, four categories—micropores, transition pores, medium pores, and large pores—were identified and quantified. The results are summarized in Table 2, where FT-I, FT-II, FT-III, and FT-IV represent micropores, transition pores, medium pores, and large pores, respectively. The proportion of each pore throat type is illustrated in Figure 20. The data show that the proportion of FT-I remains relatively constant, while FT-II increases with the number of freeze–thaw cycles. Although FT-III and FT-IV also increase with freeze–thaw cycles, the extent of their increase is smaller. Overall, a general downward trend is observed. After 12 freeze–thaw cycles, the proportion of FT-I increased by 21.01%, while FT-III and FT-IV decreased by 10.81% and 10.67%, respectively.

In this study, the micro-parameters obtained from four types of MIP tests—void ratio, permeability, characteristic length, and skewness—were statistically analyzed. The results are summarized in Table 3.

(1) Skewness

Skewness $S_{K}$ is the degree that describes the relative mean value of the distribution of the pore throat, divided into coarse skewness and fine skewness; $S_{K}=0$ for normal distribution (symmetric), $S_{K}>1$ for positive skewness (coarse skewness), and $S_{K}<1$ for negative skewness (fine skewness); its value is calculated according to the following Equation (3):

(3) $$S_{K}=\sum_{i}^{n}\Delta S_{i}{\left(R_{i}-X\right)}^{3}/100\gamma^{3}$$

(2) Permeability and Void ratio

Permeability K is describes soil’s ability to allow fluid passage under a given differential pressure. It is a parameter that characterizes the ability of the soil itself to conduct fluids.

Void ratio η is the proportion of pore space in a material, typically expressed as a percentage.

(4) K=k∆PA/μL

(5) $$\eta =V_{r}/V\times 100\%$$

In the equations above, K stands for permeability (${\mu m}^{2}$); A for liquid through the specimen cross-section (${cm}^{2}$); ∆P for liquid through the specimen before and after the pressure difference (MPa); μ for the viscosity of the fluid (mPa·s); L for the length of the specimen (cm); η for the void ratio (%); $V_{r}$ for the volume of the pore space; and V for the total volume of the specimen (${cm}^{3}$).

**Figure 21:**
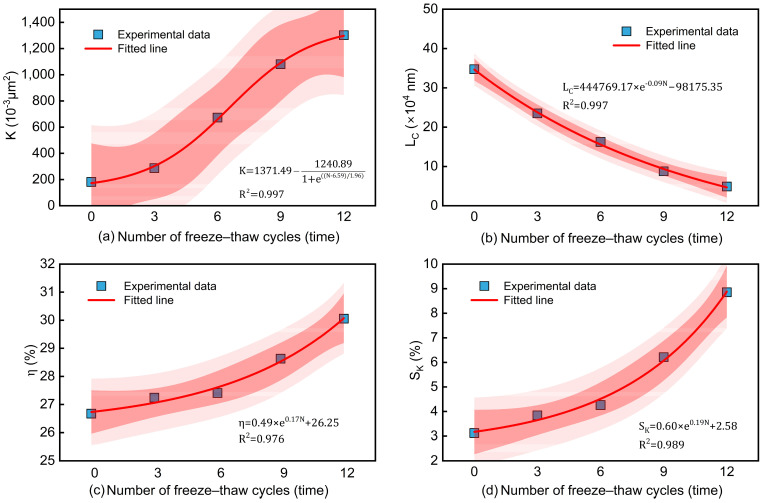
Influence of freeze–thaw cycles on parameters of waste slurry.

To investigate the effect of freeze–thaw cycles on the micro-parameters of waste slurry—void ratio, permeability, characteristic length, and skewness—and to quantify their evolution, the data were fitted as shown in Table 3. Figure 21a illustrates that the permeability of waste slurry increases exponentially with the number of freeze–thaw cycles ($R^{2}=0.997$). After 12 cycles, the permeability increased by 273.96%, nearly an order of magnitude. Figure 21b shows an exponential decline in the characteristic length with increased freeze–thaw cycles ($R^{2}=0.997$). For materials with a constant characteristic length, the properties remain stable; however, as the freeze–thaw cycles increase, the characteristic length decreases continuously. After 12 cycles, the characteristic length decreases by 44.28%, indicating ongoing changes in the specimen’s microstructure, suggesting that the freeze–thaw effect on waste slurry strength is a cumulative process.

Figure 21c,d demonstrate that the void ratio and skewness exhibited exponential growth with increased freeze–thaw cycles ($R^{2}=0.976$, $R^{2}=0.989$). The void ratio of the waste slurry increases continuously, with a 12.19% increase after 12 freeze–thaw cycles. Skewness, which represents the degree of division of pore throat distribution from the average, indicates that the pore size distribution is skewed toward larger pores. No specimen exhibited a skewness value of zero, indicates that none had a normal pore size distribution.

### 3.7. Macro–Micro Response of Mechanical Properties of Remodeled Waste Slurry Under Freeze Thaw Cycles

In this study, the Pearson correlation coefficient method was used to analyze the relationship between unconfined compressive strength, peak strength under triaxial compression, and various micro-parameters. The Pearson correlation coefficient method [35,36,37] is particularly effective for quantifying the correlation between the two sets of variables. The calculation is provided in Equation (9):

(6) $$r_{xy}=Cov\left(x,y\right)/\sqrt{\left[D\left(x\right)\cdot D\left(y\right)\right]}$$

In the equation above, $\mathrm{Cov}\left(x,y\right)$ is the covariance of x, y; $D\left(x\right)$, $D\left(y\right)$ is the variance of x, y.

In this method, the absolute value of the correlation coefficient indicates the strength of the correlation, with values closer to one signifying a stronger relationship. The degree of correlation is typically categorized as follows: high correlation ($\left|r\right|\geq 0.8$), medium correlation ($0.5\leq \left|r\right|<0.8$), low correlation ($0.3\leq \left|r\right|<0.5$), and no correlation ($\left|r\right|<0.3$). SPSS 27 statistical software was used to calculate the Pearson correlation coefficients, which were then used to establish a mathematical model via linear fitting. This model describes the relationship between the macroscopic mechanical properties and the microscopic parameters.

**Figure 22:**
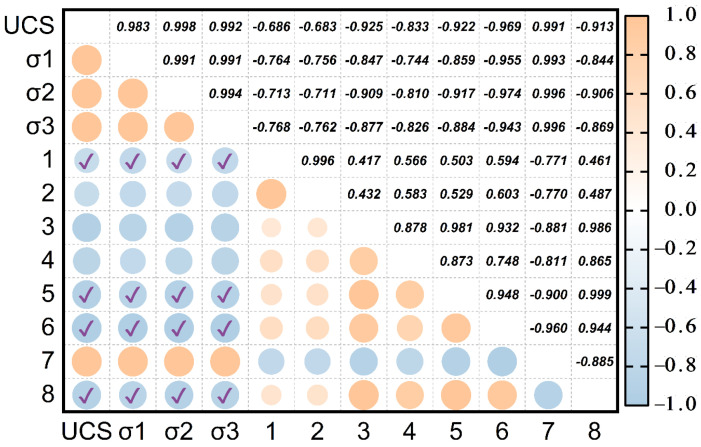
Pearson correlation of macro- and micro-parameters of waste slurry under freeze–thaw (note: 1—shape abundance; 2—circularity; 3—porosity; 4—fractal dimension; 5—void ratio; 6—permeability; 7—characteristic length; 8—skewness).

To ensure test accuracy, specimens from the same batch used for unconfined compressive strength and peak strength under triaxial circumferential pressure tests were also selected for SEM and MIP analyses. The strength values were then correlated with the SEM and MIP test parameters using Pearson’s correlation method, as shown in Figure 22.

Figure 22 illustrates that most micro-parameters are significantly correlated with macro-mechanical strength, except for the characteristic length, which exhibits a negative correlation. However, it is important to note that this correlation is not absolute. Using Pearson’s correlation coefficient method, the relationship between micro-parameters and macro-mechanical strength can be unstable due to varying circumferential pressures and freeze–thaw cycles. This instability arises from significant changes in the soil’s pore structure during freeze–thaw cycles. While qualitative analysis of unconfined strength, peak strength under triaxial circumferential pressure, and micro-parameter values is essential, a quantitative analysis is also necessary. This analysis aims to establish a mathematical model that quantitatively connects macro and micro scales.

Shape abundance (C) is defined as the ratio of the short-axis length to the long-axis length of the pores in the study area, reflecting the shape characteristics of the object in a two-dimensional plane. A low value of C indicates a indicates a more elongated shape, while a high value of C indicates a more rounded shape. The skewness of the pore throat distribution assesses whether the distribution favors coarse or fine pore throats. Permeability measures the soil’s ability to allow for fluid passage under a specific pressure difference and characterizes the waste slurry’s capability to conduct fluids. The void ratio represents the proportion of pore space in the material, reflecting the internal void ratio of the waste slurry.

The four types of micro-parameters—shape abundance, skewness, permeability, and void ratio—were selected to fit the unconfined compressive strength and peak strength value under triaxial circumferential pressure. Shape abundance and skewness characterize the pore throat size distribution as a group, while permeability and void ratio represent the proportion of internal pore space. The global optimization method [38,39] was used, which does not require an initial solution, unlike statistical analysis software such as Matlab, SAS, and SPSS. The fitting analyses revealed that the relational equations for shape abundance and skewness, permeability with void ratio and unconfined compressive strength, and peak strength under triaxial confinement pressure all satisfy the following Equation (7):

(7) $$P=m_{1}+m_{2}\cdot x+m_{3}\cdot y$$

In the equation above, P is the unconfined compressive strength or peak strength value under triaxial circumferential pressure in KPa; $m_{1}$, $m_{2}$, $m_{3}$ are constant coefficients; x is the unconfined compressive strength or peak strength value under triaxial circumferential pressure and shape abundance and skewness expressed in mathematical expressions for shape abundance, unconfined compressive strength, or peak strength value under triaxial circumferential pressure and permeability and void ratio expressed in mathematical expressions for permeability; y is the unconfined compressive strength or peak strength value under triaxial circumferential pressure and shape abundance and skewness expressed in mathematical expressions for shape abundance and skewness, unconfined compressive strength, or peak strength value under triaxial circumferential pressure and permeability and void ratio; and peak strength value under triaxial circumferential pressure and skewness in the mathematical expression for shape abundance versus skewness, unlimited compressive strength, or peak strength value under triaxial circumferential pressure and permeability versus void ratio in the mathematical expression for void ratio.

After fitting the analyzed unconfined compressive strength and shape abundance with respect to skewness, it can be expressed as

(8) $$Q_{u}=1555.91-1489.98C-15.28S_{K},R^{2}=0.922$$

The relationship between unconfined compressive strength and permeability and void ratio can be expressed as

(9) $$Q_{u}=264.87-0.92K-1.169\eta ,R^{2}=0.938$$

The relationship between peak intensity values and shape abundance and skewness under triaxial circumferential pressure can be expressed as

(10) $$\left\{\begin{array}{c} \sigma_{b1}=5268.53-5469.88C-33.34S_{K},R^{2}=0.891\\ \sigma_{b2}=4118.36-4149.26C-37.69S_{K},R^{2}=0.931\\ \sigma_{b4}=5554.20-5647.67C-36.87S_{K},R^{2}=0.927 \end{array}\right.$$

The relationship between peak intensity values and permeability and void ratio under triaxial circumferential pressure can be expressed as

(11) $$\left\{\begin{array}{c} \sigma_{b1}=-630.29-0.353K+42.01\eta ,R^{2}=0.933\\ \sigma_{b2}=-358.87-0.254K+6.16\eta ,R^{2}=0.950\\ \sigma_{b4}=-331.91-0.278K+9.74\eta ,R^{2}=0.890 \end{array}\right.\;$$

Equations (8) to (11) demonstrate that the mathematical models established for shape abundance, skewness, permeability, void ratio, and unconfined compressive strength and peak strength under triaxial circumferential compression exhibit strong correlation with $R^{2}$ values of 0.890 and 0.950, and an average value of 0.923. These strong correlations effectively reflect the macro-microcosm relationship of waste slurry under freeze–thaw cycles.

### 3.8. Microstructure Evolution of Waste Slurry During Freeze–Thaw Cycles

**Figure 23:**
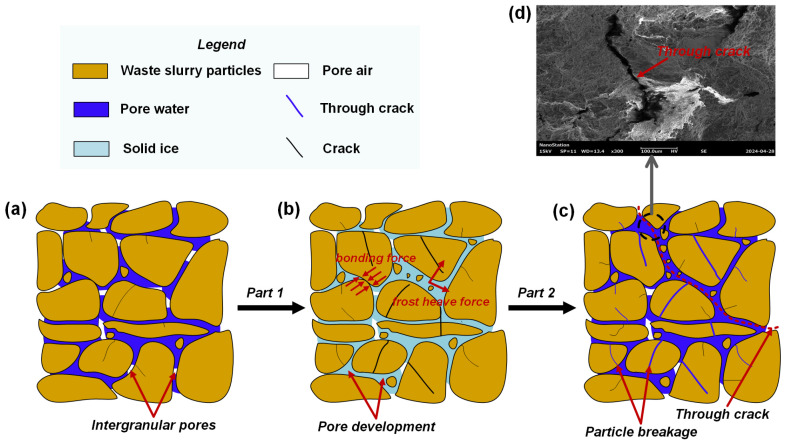
Microstructure evolution diagram of waste slurry during freeze–thaw cycle: (**a**) initial state, (**b**) frozen state, (**c**) thawed state, (**d**) SEM diagram of through crack.

As shown in Figure 23, the microstructure of waste slurry subjected to a freeze–thaw cycle can be divided into two stages. In the first stage, temperature changes and resulting tension cause water within the waste slurry to undergo phase changes, leading to the formation of fissures on the surface of primary mineral particles. In the second stage, as the phase change progresses, the water film within these fissures thickens, further widening and splitting the primary minerals. The pore structure of waste slurry is significantly affected by the freezing and expansion forces of ice crystals, as well as the bonding forces between the waste slurry particles. The freezing expansion force may exceed the bonding force between particles, causing the fragmentation of large particles into smaller ones and the collapse of the particle skeleton. Consequently, due to ice separation and expansion, the waste slurry particles undergo fragmentation and rearrangement, altering the pore morphology and size.

Freeze–thaw cycles transform micro- and mesopores within the waste slurry into macropores, as evidenced by previous studies [40,41]. Although some of the smaller particles generated during this process fill certain macropores, they are insufficient to compensate for the formation of new macropores. With an increasing number of freeze–thaw cycles, the process intensifies, leading to the gradual development and expansion of some macropores into fine microcracks, which eventually evolve into through-cracks, as observed under SEM (Figure 23d).

## 4. Conclusions

This study employed freeze–thaw cycle tests to simulate the weathering conditions encountered in the field. The mechanical properties of the waste slurry were investigated using a UU test and a UCS test. Simultaneously, the micro-deterioration mechanism of the waste slurry under freeze–thaw conditions was examined using SEM, the changes in mineral composition were analyzed by XRD, and the evolution of the microscopic pore throat structure was studied using the MIP test. The principal findings are as follows:

(1) The stress–strain curve of waste slurry under triaxial peripheral pressure exhibited four distinct stages: fissure compaction, elastic deformation, plastic yielding, and strain hardening. Peak strength was positively correlated with peripheral pressure and negatively correlated with the number of freeze–thaw cycles, indicating a strong linear relationship. The internal friction angle of the waste slurry under freeze–thaw conditions showed a wavy trend, initially increasing, then decreasing, and finally increasing again. Cohesion increased with the number of freeze–thaw cycles but eventually decreased.
(2) As the number of freeze–thaw cycles increased, the failure mode of the specimens gradually shifted from shear failure to columnar splitting and conical crushing. The stress–strain relationship of the waste slurry demonstrated a typical fissure effect under freeze–thaw action. Both unconfined compressive strength and the modulus of elasticity exhibited a linear relationship with the number of freeze–thaw cycles. The freeze–thaw coefficient of the waste slurry decreased by 52.59% after 12 cycles, indicating significant susceptibility to mechanical damage.
(3) SEM analysis revealed that the freeze–thaw environment significantly increased the pore area, size, and number of cracks in the waste slurry. This process began with particles maintaining their integrity until they reached the cracks, at which point they became detached. Additionally, the morphological distributions gradually decreased while porosity increased. XRD analysis showed no new substances were formed during the freeze–thaw cycles; only the original composition underwent micro-changes.
(4) MIP analysis indicated that the pore throat types in the waste slurry were primarily micropores, transition, medium, and large pores. The pore throat size 10nm≤e<1000nm had the largest proportion, while e<10nm had the smallest. Transition pores, accounting for 65% to 87%, were the predominant pore throat type. The four micro-parameters—void ratio, permeability, characteristic length, and skewness—demonstrated an exponential relationship with the number of freeze–thaw cycles.
(5) The correlation between macro- and micro-parameters was assessed using the Pearson correlation coefficient method, and the macro–micro response was analyzed by combining four micro-parameters: shape abundance, skewness, permeability, and void ratio. A global optimization method was employed to establish a quantitative relationship between the macro and micro scales, with the $R^{2}$ values ranging from 0.890 to 0.950. The mean $R^{2}$ value of 0.923 indicates a strong correlation between the macro properties of the waste slurry and the number of freeze–thaw cycles.

At present, some waste slurries have good engineering application effects in subgrade backfilling. However, considering the differences in freeze–thaw environments in different regions, this study is a good illustration of the use of waste slurry modifications for roadbed repair in locations where freeze–heave and thawing phenomena occur in roadbed backfill.

## Figures and Tables

**Table 1:** Basic properties of waste slurry.

Properties	Symbol	Unit	Maximum Value	Minimum Value
pH	-	-	8.29	8.21
Water Content	ω	%	26.31	25.15
Liquid Limit	$\omega_{L}$	%	34.16	30.46
Plastic Limit	$\omega_{p}$	%	19.48	17.14
Plasticity Index	$I_{p}$	%	14.68	13.32
Maximum Dry Density	$\rho_{dmax}$	$g/cm^{3}$	1.59	1.43
Optimum Water Content	$\omega_{opt}$	%	21.95	18.85

**Table 2:** Proportion of various pore throats under different freeze–thaw cycles.

Pore Throat Type	Proportion of Various Pore Throats (%)				
	FT = 0	FT = 3	FT = 6	FT = 9	FT = 12
FT-I	4.13	4.02	4.57	3.92	4.62
FT-II	65.27	67.59	70.42	70.425	86.25
FT-III	16.92	14.49	17.38	8.27	6.11
FT-IV	13.69	13.90	7.08	17.38	3.02

**Table 3:** Parameter statistics of the mercury injection method.

Number of Freeze–Thaw Cycles/Time	Micro-Parameter			
	Void Ratio/%	Permeability/${10^{-3}\mu m}^{2}$	Characteristic Length/nm	Skewness/%
0	26.67	181.12	347,089.22	3.12
3	27.24	286.99	234,896.39	3.84
6	27.41	672.54	162,197.11	4.26
9	28.63	1079.68	87,637.58	6.21
12	30.06	1302.27	48,834.41	8.85

## Data Availability

The data presented in this study are available on request from the corresponding author due to restrictions (e.g., privacy, legal or ethical reasons).
